# Vancomycin-variable enterococci as a silent reservoir of resistance: characterization of the first reported vancomycin-variable *Enterococcus faecium* isolate in Switzerland

**DOI:** 10.3389/fmicb.2026.1917481

**Published:** 2026-09-17

**Authors:** Abdessalam Cherkaoui, Patrice François, Gesuele Renzi, Nadia Gaïa, Eve-Julie Bonetti, Diego O. Andrey, Jacques Schrenzel

**Affiliations:** 1 Bacteriology Laboratory, Division of Laboratory Medicine, Department of Diagnostics, Geneva University Hospitals, Geneva, Switzerland; 2 Genomic Research Laboratory, Department of Molecular Microbiology, Faculty of Medicine, University of Geneva, Geneva, Switzerland; 3 Department of Molecular Microbiology, Faculty of Medicine, University of Geneva, Geneva, Switzerland

**Keywords:** antimicrobial susceptibility testing, *vanA* cluster, vancomycin-resistant *Enterococcus faecium*, vancomycin-variable *Enterococcus faecium*, whole-genome sequencing

## Abstract

**Objectives:**

This study reports what is, to our knowledge, the first vancomycin-variable *Enterococcus faecium* (VVEfm) isolate identified and genomically characterized in Switzerland.

**Methods:**

A vancomycin-susceptible, *vanA*-positive *E. faecium* isolate recovered from a urine culture was characterized using antimicrobial susceptibility testing (AST), *vanA/vanB* real-time PCR, population analysis profiling (PAP), spontaneous reversion assays on selective media, and whole-genome sequencing (WGS). Comparative genomic analyses were performed with five vancomycin-resistant *E. faecium* (VREfm) isolates recovered from the same patient, and three VREfm outbreak isolates from Geneva University Hospitals.

**Results:**

The VVEfm isolate exhibited vancomycin and teicoplanin minimum inhibitory concentrations (MICs) of 1.5 mg/L despite harboring the *vanA* resistance determinant. PAP showed a progressive reduction in viable counts with increasing vancomycin concentrations, although low-frequency surviving populations remained detectable at intermediate concentrations. This pattern did not demonstrate a clearly separated stable resistant subpopulation. Exposure to selective media containing vancomycin or teicoplanin induced spontaneous reversion to a resistant phenotype, with revertants displaying high-level resistance to vancomycin (MIC ≥ 256 mg/L) and teicoplanin (MIC 64 mg/L). The highest reversion frequency was observed on Brain Heart Infusion agar supplemented with 6 μg/mL vancomycin (9.5 ± 5.6 × 10^−8^). Genomic analyses demonstrated that the VVEfm isolate, its revertant, and the five VREfm isolates belonged to sequence type ST80 and formed a closely related clonal lineage differing by a maximum of four cgMLST alleles. Structural genomic analysis suggested that resistance reversion was associated with chromosomal integration of a *vanHAX*-containing mobile element adjacent to an rRNA operon, potentially restoring constitutive resistance gene expression.

**Conclusion:**

This study reports the first identification of VVEfm at Geneva University Hospitals and highlights the limitations of routine phenotypic screening methods for detecting VVE isolates. The rapid emergence of high-level glycopeptide resistance under selective pressure suggests that these isolates may act as silent reservoirs of antimicrobial resistance. Integration of molecular diagnostics with phenotypic testing and genomic surveillance is essential to improve detection, infection control, and antimicrobial stewardship strategies.

## Introduction

At least nine mechanisms of vancomycin resistance have been defined in enterococci, namely *vanA, vanB, vanC, vanD, vanE, vanG, vanL, vanM*, and *vanN* (Hawkins et al., 2024). Acquired glycopeptide resistance is predominantly mediated by the *vanA* and *vanB* gene clusters, which alter the glycopeptide binding site (Murray, 2000). The VanA phenotype is typically associated with high-level resistance to both vancomycin and teicoplanin (Murray, 2000).

The *vanA* operon comprises several genes (*vanR, vanS, vanH, vanA, vanX, vanY*, and *vanZ*) that coordinately modify the terminal sequence of peptidoglycan precursors. Specifically, the native D-Ala–D-Ala terminus is replaced by D-Ala–D-Lac, conferring high-level resistance, or more rarely by D-Ala–D-Ser, which is associated with lower-level resistance (Almeida-Santos et al., 2026). Vancomycin has an approximately 1,000-fold lower affinity for D-Ala–D-Lac than for D-Ala–D-Ala (Healy et al., 2000). The regulatory proteins VanR and VanS detect vancomycin exposure and regulate expression of the *vanHAX* operon (Merlino and Gray, 2021).

Vancomycin-variable enterococci (VVE) are enterococci that carry an acquired vancomycin-resistance determinant while remaining phenotypically susceptible to vancomycin, but that may develop a resistant phenotype under selective pressure. Most reported VVEfm isolates carry a transcriptionally silent or functionally impaired *vanA* gene cluster; however, *vanB*-positive vancomycin-susceptible *E. faecium* isolates capable of developing vancomycin resistance have also been described (Hashimoto et al., 2018; von Wintersdorff et al., 2026). Multiple mechanisms underlying this reversible phenotype have been described, including deletions involving *vanR* and *vanS*, insertion sequence-mediated disruptions, alterations affecting the *vanHAX* promoter region, deletions in *vanX* or *vanA*, and complete deletion of *vanXY* (Pinholt et al., 2015; Sivertsen et al., 2016; Wagner et al., 2021; Wagner et al., 2023).

VVEfm isolates have been reported in several geographical regions, and healthcare-associated transmission or clonal spread has been documented in multiple settings (Szakacs et al., 2014; Sivertsen et al., 2016; Hammerum et al., 2019; Wagner et al., 2023; Almeida-Santos et al., 2026; von Wintersdorff et al., 2026). In addition, within-host emergence of vancomycin resistance from a susceptible VVEfm isolate has been described during antimicrobial exposure (Coburn et al., 2014). However, the true prevalence of these organisms is likely underestimated because many microbiology laboratories rely exclusively on phenotypic susceptibility testing for VRE detection (Lee et al., 2024).

The aim of this study was therefore to describe what is, to our knowledge, the first reported and genomically characterized VVEfm isolate in Switzerland and to investigate its capacity to revert to high-level glycopeptide resistance together with the associated genomic mechanism.

## Clinical case description

A 79-year-old female patient with multiple comorbidities, including hypertension, insulin-dependent type II diabetes mellitus with complications, lower-limb arterial insufficiency, diabetic polyneuropathy, and end-stage renal disease requiring dialysis, had a history of rectal colonization with VREfm. The first VREfm-positive rectal screening sample was recorded on July 27, 2021, and VREfm carriage was subsequently documented on more than seven occasions through December 8, 2021. This was followed by a period during which no VREfm was detected. In April 2025, the patient presented with wet necrosis of the right heel. *E. faecium* isolates, *vanA/vanB*-negative, vancomycin-susceptible were recovered from four intraoperative samples collected on April 2, 2025, and from two samples collected from the right iliac fossa on April 10, 2025. The infection was managed surgically and with antimicrobial therapy consisting of amoxicillin–clavulanic acid, followed by amoxicillin and doxycycline.

VREfm was again detected from July 31, 2025 onward, with several rectal or stool samples confirming carriage. The isolates recovered between July and December 2025 were phenotypically resistant to vancomycin and carried *vanA* but not *vanB*. On December 15, 2025, a urine culture yielded *Proteus mirabilis* at 10^5^ CFU/mL, *Klebsiella aerogenes* at 10^5^ CFU/mL, and *E. faecium* at 10^3^ CFU/mL. The *E. faecium* isolate was phenotypically susceptible to vancomycin, but real-time PCR detected *vanA*, leading to its classification as VVEfm.

The available clinical and microbiological data do not allow definitive distinction among persistent low-level carriage, recolonization, or within-host evolution. The chronological sequence of events is summarized in Table 1.

**Table 1:** Chronology of key microbiological findings and antimicrobial exposures.

Date/period	Specimen or clinical event	Microbiological finding	Phenotype	*vanA*	*vanB*	Antimicrobial exposure
27 July 2021–8 December 2021	Repeated rectal screening samples	VREfm carriage documented on more than seven occasions	Vancomycin-resistant	Positive	Negative	Not reported
9 December 2021–1 April 2025	Surveillance period	No detectable VREfm carriage				Not reported
2 April 2025	Four intraoperative samples from right heel infection	*E. faecium* recovered	Vancomycin-susceptible	Negative	Negative	Amoxicillin–clavulanic acid, followed by amoxicillin and doxycycline
10 April 2025	Two samples from right iliac fossa	*E. faecium* recovered	Vancomycin-susceptible	Negative	Negative	Amoxicillin–clavulanic acid, followed by amoxicillin and doxycycline
31 July 2025	Rectal swab	Isolate 6, VREfm	Vancomycin-resistant	Positive	Negative	Not reported
7 August 2025	Rectal swab	Isolate 5, VREfm	Vancomycin-resistant	Positive	Negative	Not reported
19 August 2025	Rectal swab	Isolate 4, VREfm	Vancomycin-resistant	Positive	Negative	Not reported
5 November 2025	Stool	Isolate 3, VREfm	Vancomycin-resistant	Positive	Negative	Not reported
7 December 2025	Stool	Isolate 2, VREfm	Vancomycin-resistant	Positive	Negative	Not reported
15 December 2025	Urine	Isolate 1, VVEfm; mixed culture also containing *P. mirabilis* and *K. aerogenes*	Vancomycin-susceptible	Positive	Negative	Not reported

## Materials and methods

### Culture work-up

Urine cultures and VRE screening were performed according to previously validated protocols (Cherkaoui et al., 2019; Cherkaoui and Schrenzel, 2022; Cherkaoui et al., 2026). Briefly, the workflow combined total laboratory automation (WASPLab, Copan) with artificial intelligence-assisted image analysis software (PhenoMATRIX™ PLUS) and chromogenic media (CHROMID® VRE agar and CHROMID® CPS ELITE). Species identification was performed by matrix-assisted laser desorption ionization–time of flight mass spectrometry (MALDI-TOF MS; Bruker Daltonics, Bremen, Germany). For the presumptive VRE colonies recovered on CHROMID® VRE agar, species identification by MALDI-TOF MS and phenotypic antimicrobial susceptibility testing (AST) were systematically combined with *vanA/vanB* real-time PCR.

### Antimicrobial susceptibility testing

Antimicrobial susceptibility testing was performed using a fully automated disk diffusion system (Radian, Copan) (Cherkaoui et al., 2021). Inhibition-zone diameters and MIC values were interpreted according to the European Committee on Antimicrobial Susceptibility Testing clinical breakpoint tables, version 15.0, 2025. Species-specific criteria for *Enterococcus* spp. were applied where available. Vancomycin, teicoplanin, and daptomycin MICs were determined using gradient diffusion strips (Etest®, bioMérieux) according to the manufacturer’s instructions. No EUCAST clinical breakpoint was available for daptomycin against *Enterococcus faecium*. Daptomycin results were therefore reported as MIC values only.

### Spontaneous VVEfm-to-RVVEfm reversion frequency

Colonies of the VVEfm isolate, confirmed by AST and *vanA/vanB* real-time PCR, were used to prepare bacterial suspensions adjusted to 0.5, 1, 2, and 3 McFarland standards. For each suspension, viable bacterial counts were determined by serial dilution and plating on columbia agar. Plates were incubated overnight at 37 °C, and colonies were enumerated to calculate the viable inoculum in CFU/mL. Aliquots of 10 μL and 100 μL from each inoculum were plated in duplicate onto Mueller–Hinton agar containing 5 mg/L teicoplanin (MHT5), Brain Heart Infusion agar supplemented with 6 mg/L vancomycin (BHIV), and CHROMID® VRE agar. Selective plates were incubated at 37 °C for 72 h. Reversion frequency was calculated independently for each inoculum, inoculated volume, selective medium, and replicate according to the following formula: number of reverted colonies divided by the total number of viable cells plated. The denominator was calculated from the viable count of the corresponding inoculum multiplied by the volume plated. The reported mean reversion frequency and standard deviation were calculated.

### Population analysis profiling

Population analysis profiling was performed using a modified spotting method as previously described by Pfeltz et al. (2001). Four independent biological cultures of the VVEfm isolate were analyzed. Each culture was adjusted to a 2.0 McFarland standard and serially diluted 10-fold up to 10^−7^. Aliquots of 10 μL from the appropriate dilutions were inoculated onto Mueller–Hinton agar containing vancomycin at 0.25, 0.5, 1, 2, 4, 8, 16, 32, and 64 mg/L. Colonies were counted after 24 h of incubation at 37 °C. Viable counts are expressed as log10 CFU/mL.

### Whole-genome sequencing and bioinformatics analyses

Genomic DNA was extracted using DNeasy columns (Qiagen). High-throughput sequencing was performed on an Illumina NovaSeq 6000 platform (Illumina, San Diego, CA, USA). Read quality was assessed using FastQC and filtered with Trimmomatic v0.39. Genome assembly was performed using SPAdes v3.15.5 with the parameters—k 21, 33, 55, 77, 99 and –careful. Genome annotation was conducted using Prokka v1.10. Sequence data from the VVEfm and revertant RVVEfm isolates were deposited in the European Nucleotide Archive under accession number PRJEB112819. Antimicrobial resistance genes were identified using tools provided by the Center for Genomic Epidemiology (CGE). Phylogenetic relationships among isolates were investigated using SNP-based methods implemented in CSI Phylogeny and the cloud-based bioinformatics platform 1928 Diagnostics (version, 2026-05.3).

All corresponding quality metrics and genomic data are comprehensively summarized in Supplementary Tables S1, S2.

## Results

### Antimicrobial susceptibility testing

Tables 2, 3 summarize the antimicrobial susceptibility profiles of the VVEfm isolate and the five VREfm isolates recovered from our patient. Results are presented together with categorical interpretations where applicable. A zone diameter of 6 mm indicated absence of a measurable inhibition zone beyond the disk diameter. Aside from susceptibility to vancomycin and teicoplanin, the antimicrobial susceptibility profiles were comparable among all isolates. All isolates displayed comparable tigecycline and linezolid susceptibility profiles. The daptomycin MIC was 2 mg/L for all isolates; this value is reported quantitatively without categorical interpretation because no applicable EUCAST clinical breakpoint was available for daptomycin against *E. faecium* under the guideline version used. The VVEfm isolate exhibited vancomycin and teicoplanin MICs of 1.5 mg/L, whereas the five VREfm isolates demonstrated markedly elevated MICs of ≥256 mg/L for both glycopeptides.

**Table 2:** Antimicrobial susceptibility profile of the VVEfm isolate recovered from our patient.

Antimicrobial agent	Disk content (μg)	Zone diameter (mm)	MIC method	MIC (mg/L)	Interpretation
Ampicillin	2	6	Not performed	ND	R
Gentamicin	30	6	Not performed	ND	R
Tigecycline	15	25	Not performed	ND	S
Linezolid	10	23	Not performed	ND	S
Vancomycin	5	19	Gradient diffusion	1.5	S
Teicoplanin	30	17	Gradient diffusion	1.5	S
Daptomycin	NA	ND	Gradient diffusion	2	

MICs and inhibition-zone diameters were interpreted according to EUCAST clinical breakpoint tables, version 15.0 (2025), using criteria applicable to *Enterococcus* spp. Daptomycin MIC values are reported quantitatively without categorical interpretation because no applicable EUCAST clinical breakpoint was available for *E. faecium*. Daptomycin MICs were performed using Etest® gradient diffusion strips according to the manufacturer’s instructions.

A zone diameter of 6 mm indicated absence of a measurable inhibition zone beyond the disk diameter.

ND, Not determined; NA, Not applicable.

**Table 3:** Antimicrobial susceptibility profiles of the five VREfm isolates recovered from our patient.

Antimicrobial agent	Disk content (μg)	Zone diameter (mm)	MIC method	MIC (mg/L)	Interpretation
Ampicillin	2	6	Not performed	ND	R
Gentamicin	30	6	Not performed	ND	R
Tigecycline	15	25	Not performed	ND	S
Linezolid	10	23	Not performed	ND	S
Vancomycin	5	6	Gradient diffusion	≥256	R
Teicoplanin	30	6	Gradient diffusion	≥256	R
Daptomycin	NA	ND	Gradient diffusion	2	

### Spontaneous VVEfm-to-RVVEfm reversion frequency

Table 4 presents the distribution of RVVEfm revertants recovered on the three selective media tested, namely MHT5, BHIV, and CHROMID® VRE agar. All revertant isolates displayed high-level resistance to vancomycin (MIC ≥ 256 mg/L) and teicoplanin (MIC = 64 mg/L; Table 5). BHIV yielded the highest number of revertants, with a reversion frequency of 9.5 ± 5.6 × 10^−8^. The RVVEfm colonies were subsequently subjected to extended AST and whole-genome sequencing.

**Table 4:** Distribution of RVVEfm revertant isolates across the three selected culture media and four inoculum concentrations tested.

McFarland inoculum	Volume plated (mL)	Selective medium	Replicate	Viable count of inoculum (CFU/mL)	Total viable CFU plated	Colonies recovered	Colonies confirmed as RVVEfm	Reversion frequency	Reported mean reversion frequency	Standard deviation
0.5	0.01	MHT5	2	1.80E+08	1.80E+06	0	0		3.5E−08	1.9E−08
0.5	0.1	MHT5	2	1.80E+08	1.80E+07	1	1	5.6E−08		
1	0.01	MHT5	2	3.20E+08	3.20E+06	0	0			
1	0.1	MHT5	2	3.20E+08	3.20E+07	1	1	3.1E−08		
2	0.01	MHT5	2	5.90E+08	5.90E+06	0	0			
2	0.1	MHT5	2	5.90E+08	5.90E+07	1	1	1.7E−08		
3	0.01	MHT5	2	9.10E+08	9.10E+06	0	0			
3	0.1	MHT5	2	9.10E+08	9.10E+07	0	0			
0.5	0.01	BHIV	2	1.80E+08	1.80E+06	0	0		9.5E−08	5.6E−08
0.5	0.1	BHIV	2	1.80E+08	1.80E+07	0	0			
1	0.01	BHIV	2	3.20E+08	3.20E+06	0	0			
1	0.1	BHIV	2	3.20E+08	3.20E+07	3	3	9.4E−08		
2	0.01	BHIV	2	5.90E+08	5.90E+06	1	1	1.7E−07		
2	0.1	BHIV	2	5.90E+08	5.90E+07	5	5	8.5E−08		
3	0.01	BHIV	2	9.10E+08	9.10E+06	0	0			
3	0.1	BHIV	2	9.10E+08	9.10E+07	3	3	3.3E−08		
0.5	0.01	CHROMID® VRE agar	2	1.80E+08	1.80E+06	0	0		2.5E−08	1.1E−08
0.5	0.1	CHROMID® VRE agar	2	1.80E+08	1.80E+07	0	0			
1	0.01	CHROMID® VRE agar	2	3.20E+08	3.20E+06	0	0			
1	0.1	CHROMID® VRE agar	2	3.20E+08	3.20E+07	0	0			
2	0.01	CHROMID® VRE agar	2	5.90E+08	5.90E+06	0	0			
2	0.1	CHROMID® VRE agar	2	5.90E+08	5.90E+07	1	1	1.7E−08		
3	0.01	CHROMID® VRE agar	2	9.10E+08	9.10E+06	0	0			
3	0.1	CHROMID® VRE agar	2	9.10E+08	9.10E+07	3	3	3.3E−08		

Reversion frequency was calculated as the number of confirmed RVVEfm colonies divided by the total number of viable VVEfm CFU plated. Viable counts were determined in parallel on non-selective agar.

MHT5, Mueller–Hinton agar containing 5 mg/L teicoplanin; BHIV, Brain Heart Infusion agar supplemented with 6 mg/L vancomycin; CHROMID® VRE agar (BioMérieux).

**Table 5:** Antimicrobial susceptibility profile of the RVVEfm revertant isolate.

Antimicrobial agent	Disk content (μg)	Zone diameter (mm)	MIC method	MIC (mg/L)	Interpretation
Ampicillin	2	6	Not performed	ND	R
Gentamicin	30	6	Not performed	ND	R
Tigecycline	15	26	Not performed	ND	S
Linezolid	10	26	Not performed	ND	S
Vancomycin	5	6	Gradient diffusion	≥256	R
Teicoplanin	30	6	Gradient diffusion	64	R
Daptomycin	NA	ND	Gradient diffusion	2	

### Population analysis profiling

The PAP results for the VVEfm isolate are shown in Figure 1. The four biological replicates displayed broadly consistent growth patterns. Increasing vancomycin concentrations were associated with a progressive decline in viable bacterial counts, with the most pronounced reduction occurring between approximately 0.5 and 1 mg/L. Small surviving populations, generally in the range of 10^2^–10^3^ CFU/mL, remained detectable between 1 and 4 mg/L, whereas no growth was observed at 8 mg/L. These findings indicate the presence of low-frequency surviving subpopulations under vancomycin exposure. However, the PAP pattern did not demonstrate a clearly separated, stable resistant subpopulation characteristic of classical heteroresistance.

**Figure 1:**
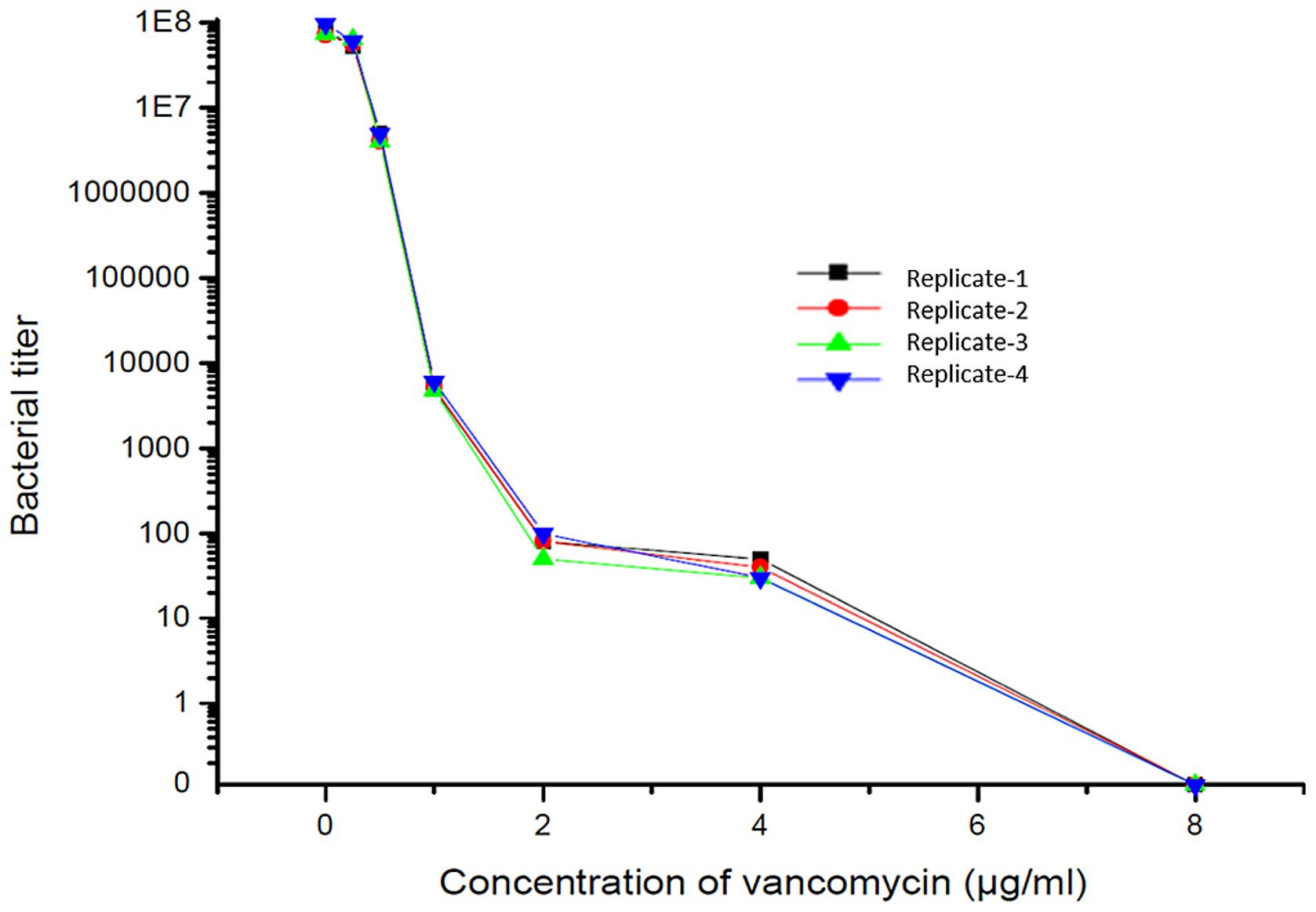
Population analysis profile of the VVEfm isolate under increasing vancomycin concentrations. Four biological replicates were tested. Viable counts were calculated from serially diluted cultures plated in 10 μL aliquots and are expressed as log10 CFU/mL.

### Core-genome MLST and SNP analysis

The VVEfm isolate, its RVVEfm revertant, and the five VREfm isolates were assigned to sequence type ST80. Pairwise comparison showed a maximum distance of four cgMLST alleles, supporting close relatedness among the seven isolates (Figure 2A). Whole-genome SNP analysis identified several subclusters within this closely related group. Isolate pairs VRE2/VRE4 and VRE5/VRE6 showed the shortest pairwise SNP distances. The RVVEfm isolate occupied a relatively longer branch than expected from the cgMLST analysis (Figure 2B). However, its precise position should be interpreted cautiously because cgMLST and SNP analysis evaluate different categories of genomic variation and may be differently influenced by reference coverage, mobile genetic elements, and recombination. Consequently, the relatively longer RVVEfm branch in the SNP tree does not necessarily contradict the close relationship indicated by the maximum four-allele cgMLST distance. The study isolates were distinct from the ST80 VREfm isolates associated with recent outbreaks at our institution (Figures 3A,B).

**Figure 2:**
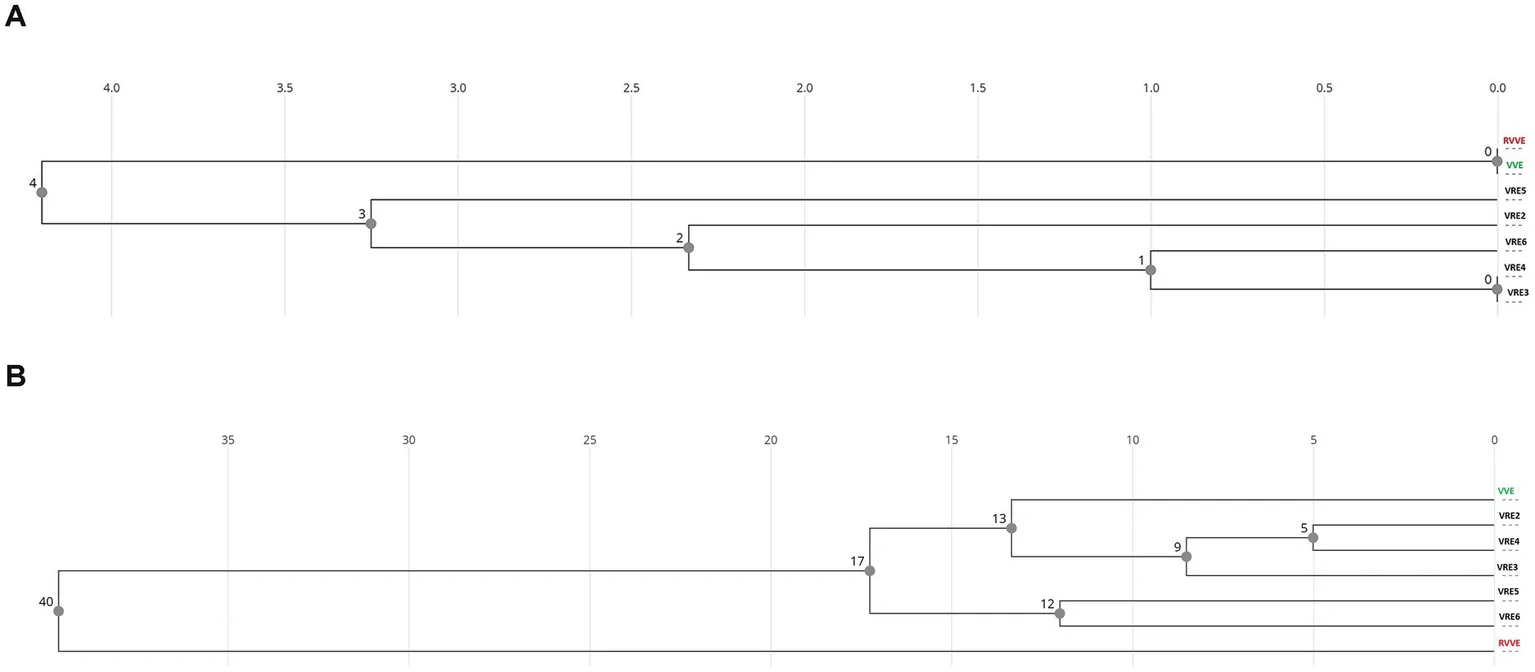
Genomic relatedness among the VVEfm isolate, the experimentally derived RVVEfm revertant, and five VREfm isolates recovered from the same patient. **(A)** Core-genome multilocus sequence typing analysis. Branching and pairwise distances reflect differences in called cgMLST alleles. **(B)** Whole-genome SNP-based phylogeny. All isolates belonged to sequence type ST80. VVEfm, vancomycin-variable *Enterococcus faecium*; RVVEfm, reverted vancomycin-variable *E. faecium* with restored glycopeptide resistance; VREfm, vancomycin-resistant *E. faecium*; cgMLST, core-genome multilocus sequence typing; SNP, single-nucleotide polymorphism.

**Figure 3:**
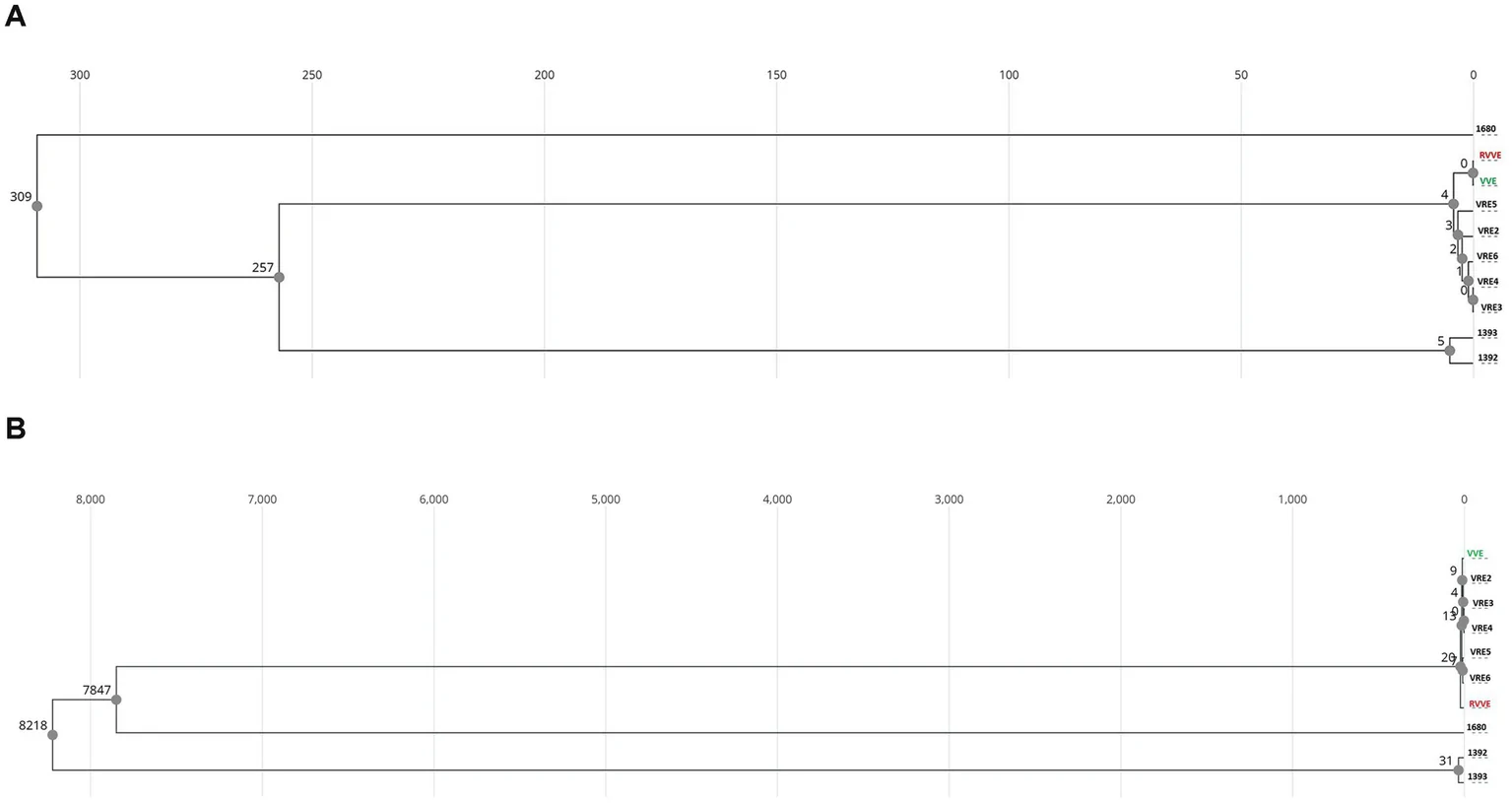
Genomic comparison of the patient-associated VVEfm, RVVEfm, and VREfm isolates with recent ST80 VREfm outbreak isolates from Geneva University Hospitals. **(A)** Core-genome multilocus sequence typing analysis. Patient-associated isolates and institutional outbreak comparator isolates are identified by separate metadata groups. Branching and pairwise distances represent cgMLST allelic differences. **(B)** Whole-genome SNP phylogeny. The patient-associated isolates form a cluster distinct from the recent institutional outbreak isolates.

### Spontaneous VVEfm-to-RVVEfm reversion mechanism

Analysis of the VVEfm and RVVEfm genomes revealed that the revertant assembly contains a fused contig in which chromosomal DNA is joined to an approximately 33 kb segment derived from the VVEfm mobile vancomycin-resistance element. Figure 4 represents a schematic comparison of the parental VVEfm and the reverted high-level glycopeptide-resistant derivative (RVVEfm). In the parental VVEfm draft assembly, the *vanA*-containing mobile resistance region includes the regulatory genes *vanR* and *vanS*, the resistance-associated *vanHAX* genes, and the accessory genes *vanY* and *vanZ*. The isolate remained phenotypically susceptible to vancomycin (MIC 1.5 mg/L). In the RVVEfm assembly, an approximately 33 kb *vanHAX*-containing segment derived from the parental mobile resistance element was identified in an inferred new chromosomal context. This rearranged region was inferred to be adjacent to an rRNA operon and was associated with restoration of high-level glycopeptide resistance. A plasmid-derived remnant contig was also identified in the RVVEfm assembly. The proposed rearrangement is consistent with a structural mechanism of resistance reversion rather than a simple corrective point mutation within the core *vanHAX* genes. This genomic rearrangement likely places the resistance genes under the influence of strong constitutive transcription, thereby bypassing the defective *vanR/vanS*-mediated induction system present in the parental VVEfm strain. This observation is consistent with the VVE-to-RVVE reversion mechanism previously described in OI25-like strains (McInnes et al., 2024). In the parental VVE strain, *vanHAX* expression is impaired because of defects in the regulatory region surrounding *vanR* and its promoter. In contrast, revertant strains restore high-level glycopeptide resistance through integration of the *vanHAX*-containing element downstream of a ribosomal RNA (*rRNA*) operon, resulting in strong constitutive co-transcription of the resistance genes (McInnes et al., 2024).

**Figure 4:**
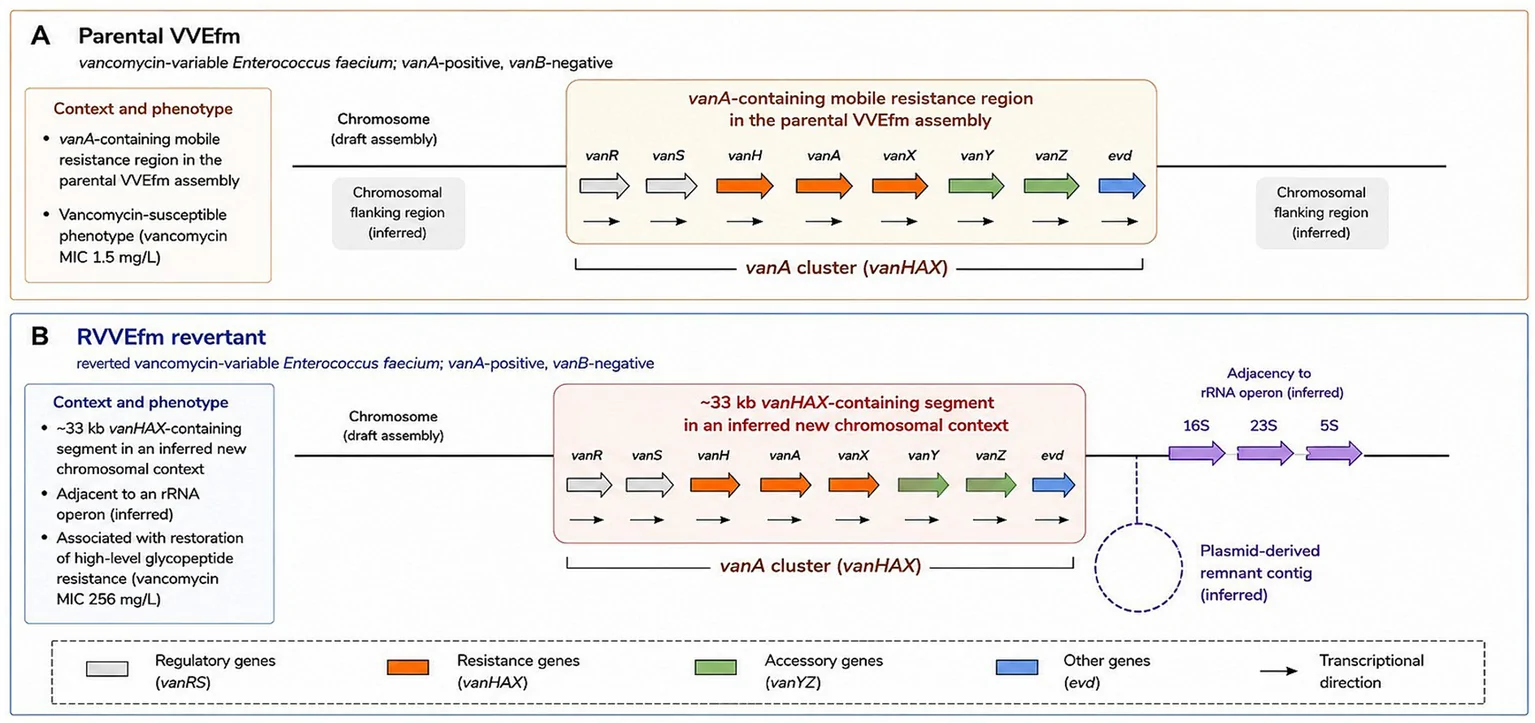
Inferred genomic rearrangement associated with reversion from VVEfm to RVVEfm. **(A)** Parental VVEfm draft assembly, *vanA*-containing mobile resistance region identified in the draft assembly together with the vancomycin-susceptible phenotype. **(B)** RVVEfm assembly, an approximately 33 kb *vanHAX*-containing segment derived from the parental mobile resistance element was identified in an inferred new chromosomal context. This figure is inferred from Illumina short-read draft genome assemblies and that the exact insertion boundaries, orientation, and complete architecture of the rearranged region would require confirmation by long-read sequencing.

## Discussion

Vancomycin-resistant enterococci have emerged as a major healthcare-associated pathogen in Switzerland, with recent outbreaks driven by the dissemination of epidemic clones. Between 2018 and 2024, the ST612 clone, frequently associated with reduced susceptibility to daptomycin, caused multiple outbreak clusters across the country (Vuichard-Gysin et al., 2025). These isolates carried mutations in the *liaR* and *liaS* genes, which are involved in cell envelope stress responses and may contribute to reduced daptomycin susceptibility (Vuichard-Gysin et al., 2025). The clone was identified in different Swiss cantons, suggesting previously unrecognized transmission networks and substantial inter-cantonal spread (Vuichard-Gysin et al., 2025). Another major outbreak, occurring between 2017 and 2020, was caused by the ST796 clone, which spread rapidly among hospitals in the Canton of Bern (Wassilew et al., 2018). More recently, a nationwide genomic surveillance study demonstrated the emergence and dissemination of VREfm ST80 and ST612 clones across Switzerland, confirming ongoing transmission between cantonal healthcare networks, and showing that the broader ST80 lineage contains several sub-lineages (Blanc et al., 2026). Although Switzerland historically maintained a relatively low prevalence of VRE, rates have increased in recent years, rising from approximately 1.7% in 2020 to 2.2% in 2023 (Blanc et al., 2026).

VVE strains represent a particular diagnostic and epidemiological challenge because they carry an acquired vancomycin-resistance determinant, most commonly *vanA* but also, in some reports, *vanB*, while remaining phenotypically susceptible to vancomycin (Hashimoto et al., 2018). These isolates may subsequently develop resistance under antibiotic selective pressure through mechanisms that depend on the underlying van genotype and regulatory defect (Hawkins et al., 2024).

Epidemiological studies from multiple countries have reported increasing prevalence of VVE in hospital settings, particularly among critically ill and immunocompromised patients (Coburn et al., 2014; Szakacs et al., 2014). Several outbreak investigations have demonstrated that VVE dissemination is largely driven by clonal transmission within healthcare institutions (Sivertsen et al., 2016; Hammerum et al., 2019; Wagner et al., 2023). Recent evidence suggests that VVE strains are substantially underdetected because routine laboratory screening strategies primarily rely on phenotypic resistance detection. In a Canadian study, nearly half of all *vanA*-positive isolates were identified as VVE strains, highlighting the diagnostic and infection-control challenges posed by these organisms (Kohler et al., 2018). Similarly, genomic analyses from Italy demonstrated that VVE strains may acquire vancomycin resistance through mutations affecting the *vanHAX* regulatory region under antibiotic selective pressure (Coccitto et al., 2024). In Denmark, VVE ST1421-CT1134 became the dominant *vanA*-positive *E. faecium* clone by 2019 and disseminated across all five healthcare regions (Hammerum et al., 2019). Collectively, these findings indicate that VVE epidemiology is characterized by increasing hospital prevalence, clonal dissemination, substantial genomic plasticity, and rapid acquisition of resistance during therapy. Standard phenotypic methods, including disk diffusion and conventional MIC testing, are unable to identify VVE strains because these organisms remain phenotypically susceptible to vancomycin. Reported vancomycin MIC values for VVE isolates are generally ≤2 mg/L, whereas revertant strains often exhibit MICs ≥256 mg/L (Wagner et al., 2023). Consequently, chromogenic screening media may fail to detect VVE strains because the selective pressure applied during routine culture and susceptibility testing is insufficient to induce resistance-conferring genomic rearrangements (von Wintersdorff et al., 2026).

Although the circulation of phenotypically resistant VREfm clones in Switzerland has been increasingly documented, we found no previous peer-reviewed report specifically describing a *vanA/vanB*-positive, vancomycin-susceptible isolate in the country. Thus, to our knowledge, the isolate described here represents the first reported and genomically characterized VVEfm case in Switzerland. This distinction should nevertheless be interpreted cautiously, as VVEfm isolates may have circulated undetected because routine rectal screening relies on selective culture methods that may not recover phenotypically vancomycin-susceptible isolates.

## Conclusion

This case highlights the limitations of routine phenotypic susceptibility testing and chromogenic screening methods, which may fail to detect *vanA/vanB*-positive, vancomycin-susceptible enterococci. Consequently, VVE strains may remain unrecognized and silently contribute to the dissemination of glycopeptide resistance within healthcare settings. The integration of molecular detection methods targeting *vanA/vanB* together with conventional AST is therefore essential for accurate identification of the VVE isolates. Given the increasing circulation of epidemic VRE clones in Switzerland and internationally, enhanced surveillance strategies incorporating genomic analyses are needed to better understand the epidemiology, transmission dynamics, and clinical impact of VVE. Early recognition of these isolates is critical to support infection prevention and antimicrobial stewardship efforts.

## Data Availability

The datasets presented in this study can be found in online repositories. The names of the repository/repositories and accession number(s) can be found in the article/Supplementary material.
